# Multizone Paper Platform for 3D Cell Cultures

**DOI:** 10.1371/journal.pone.0018940

**Published:** 2011-05-06

**Authors:** Ratmir Derda, Sindy K. Y. Tang, Anna Laromaine, Bobak Mosadegh, Estrella Hong, Martin Mwangi, Akiko Mammoto, Donald E. Ingber, George M. Whitesides

**Affiliations:** 1 Department of Chemistry and Chemical Biology, Harvard University, Cambridge, Massachusetts, United States of America; 2 Wyss Institute for Biologically Inspired Engineering, Harvard University, Cambridge, Massachusetts, United States of America; 3 Vascular Biology Program, Children's Hospital and Harvard Medical School, Boston, Massachusetts, United States of America; 4 School of Engineering and Applied Sciences, Harvard University, Cambridge, Massachusetts, United States of America; Massachusetts Institute of Technology, United States of America

## Abstract

*In vitro* 3D culture is an important model for tissues *in vivo*. Cells in different locations of 3D tissues are physiologically different, because they are exposed to different concentrations of oxygen, nutrients, and signaling molecules, and to other environmental factors (temperature, mechanical stress, etc). The majority of high-throughput assays based on 3D cultures, however, can only detect the *average* behavior of cells in the whole 3D construct. Isolation of cells from specific regions of 3D cultures is possible, but relies on low-throughput techniques such as tissue sectioning and micromanipulation. Based on a procedure reported previously (“cells-in-gels-in-paper” or CiGiP), this paper describes a simple method for culture of arrays of thin planar sections of tissues, either alone or stacked to create more complex 3D tissue structures. This procedure starts with sheets of paper patterned with hydrophobic regions that form 96 hydrophilic zones. Serial spotting of cells suspended in extracellular matrix (ECM) gel onto the patterned paper creates an array of 200 micron-thick slabs of ECM gel (supported mechanically by cellulose fibers) containing cells. Stacking the sheets with zones aligned on top of one another assembles 96 3D multilayer constructs. De-stacking the layers of the 3D culture, by peeling apart the sheets of paper, “sections” all 96 cultures at once. It is, thus, simple to isolate 200-micron-thick cell-containing slabs from each 3D culture in the 96-zone array. Because the 3D cultures are assembled from multiple layers, the number of cells plated initially in each layer determines the spatial distribution of cells in the stacked 3D cultures. This capability made it possible to compare the growth of 3D tumor models of different spatial composition, and to examine the migration of cells in these structures.

## Introduction

The culture of isolated cells *in vitro* makes it possible to study aspects of cell and organismic (specifically human) biology, and can contribute to techniques for the development of drugs. To date, the majority of cell-based assays have been conducted using cells that grow as 2D monolayers on the surface of polymer or glass dishes. In this non-physiological environment, many cell types develop phenotypes very different from cells *in vivo* [1]. Cells *in vitro*, however, can develop morphology and physiology similar to that of analogous cell types *in vivo* when they are cultured as 3D aggregates, or as suspensions inside hydrogels composed of extracellular matrix (ECM) proteins [2]–[8].

Three classes of environmental factors contribute to the differences between cells in 3D-cultures and 2D monolayers: (i) Cells in 3D experience a series of polarizing chemical cues that are entirely different from those in 2D cultures. Spatial differences in the composition of the extracellular space that surrounds the cells influence both the distribution of cell-cell and cell-matrix contacts on the surface of the cells, and the distribution of biomolecules inside the cells. These changes in the polarity of cells have pronounced effects on cell signaling [9]–[11]. (ii) Cells modulate their mechanical properties and physiology in response to the mechanical properties of their environment. The distribution of strain in cells growing on the static, rigid 2D substrate of a culture dish is largely irrelevant to that of cells *in vivo* that are surrounded by a three-dimensional environment [7], [12]–[14]. (iii) Mass transport influences the access of cells to O_2_, nutrients, and to various soluble factors [15]–[17]. Molecular gradients, however, are largely absent in the cells growing in a monolayer in convectively stirred media.

Because the distributions of nutrients, waste products, and signaling molecules are non-uniform in the extracellular space in 3D culture and *in vivo*, the behavior of cells in different areas of a 3D culture differs. For example, the partial pressure of oxygen (P_O2_) in 3D culture changes from 120 mm Hg for cells in direct contact with oxygenated culture medium, to P_O2_∼0 in cells located a few hundred microns inside the culture [2], [18], [19]. Normoxic and hypoxic cells in these two locations differ in their proliferative capacity, gene expression profile, physiology, and ability to respond to many chemical and physical stimuli. Because the concentration of oxygen inside 3D culture changes on the length scale of a few hundreds of microns, 3D cell-based assays should have the capability to analyze cells inside the tissue with spatial resolution of at least 100 microns.

### What are the problems with the existing methods?

Assays using 96- and 384-well plates are the standards for cell-based assays in both fundamental and pharmaceutical research; they serve as the basis of many high-throughput assays. Among the types of 3D cultures that use 96-well formats are those that assay cell aggregates, or cells in gel particles, suspended in wells, and those that assay cells grown in thin gels on the surface of 96-well plates or 96-well inserts [20], [21]. The majority of these methods use 3D constructs that are typically non-uniform in dimensions. Exposure of cells in these constructs to O_2_ and to other factors is thus also non-uniform. These assays can provide information about the average behavior of cells in 3D, but they fail to provide information about cells in different areas within a single 3D construct. Platforms based on microfluidic technologies have finer control over the parameters dictating the cellular microenvironments (for reviews see [22]–[24]); but these methods are typically difficult to apply to parallel testing of multiple culture conditions.

Analysis of cells in different regions of a 3D construct-for example, a gel particle having dimensions of a few hundred microns-is possible using confocal imaging [25], [26] but it may be slow and technically challenging. Biochemical processing of cells (e.g immunostaining) cannot be performed in intact 3D tissues because high-molecular-weight reagents, such as antibodies, diffuse into the constructs slowly [27]. The use of reagents based on enzymatic reactions (e.g., metabolic probes) is further complicated by reaction-diffusion phenomena in 3D [28], [29]. Staining and biochemical characterizations, therefore, usually require physical isolation of cells from different regions of the 3D construct [30]. Techniques currently used for physical isolations (e.g. microtome, laser capture microdissection, digestion and sorting [31]) are inherently low-throughput and disruptive to cells.

### How do we create and analyze 3D cultures of controlled geometry? Cells in gels in paper (CiGiP)

To facilitate the rapid isolation of live cells from 3D cultures, we have developed a procedure that assembles 3D tissues by stacking multiple layers of paper that support ECM-derived hydrogel slabs containing suspensions of cells. Paper provides mechanical support for thin, mechanically fragile hydrogels, and it does not interfere with the proliferation of cells [28], [32]–[34]. It is simple to separate layers of paper permeated with gel by peeling them apart gently, and to examine cells in individual layers of the multi-layer constructs [28]. The thickness of each “section” is that of the paper (200 micron or less). These values are less than the penetration depth of oxygen into metabolically active tissue, and therefore cells in a single layer of paper are not limited in growth or metabolism. This manuscript extends a previously developed technology [28], and demonstrates that rapid generation and analysis of 3D cultures can be achieved by stacking sheets of paper that contain 96 cell-containing zones.

### Experimental design

There are four requirements for the successful design of the platform that supports 96-zone multilayer cultures: (i) Plating cells on each 96-zone layer must be compatible with high-throughput liquid handling (i.e., delivery of cells should be possible by simple serial spotting within one 2D plane). (ii) Within each layer, cells must be restricted to their zone. Cell-containing areas must be separated by cell-free and cell-impermeable areas. (iii) Upon stacking of 96-zone sheets, all zones must be aligned and be in vertical contact to allow cells in each zone to interact with cells in the same zone from the adjacent layers. (iv) Assembly and analysis of 96-zone layers must be sufficiently simple that it can be conducted in any biological laboratory without expensive or complex equipment.

This paper describes the design and development of the tools and infrastructure that satisfy these requirements, to enable parallel culture and rapid analysis of 96 3D cultures in multiple stacked layers of paper. We demonstrate that multi-zone, multi-layer culture makes it possible to generate 96 multi-layer 3D cultures simultaneously using a single step of stacking. A single de-stacking step-peeling apart the layers of cell-containing paper-isolates cells from different regions of all 96 cultures at once. The “sectioned layers” can be analyzed using standard 2D imaging techniques. We demonstrate the characteristics of this platform with a series of cell-based assays. We also suggest the types of cell-based assay that can be performed, with further development, using this platform.

## Results

### Preparation of the paper substrates for 96-zone 3D cultures

#### Choice of paper

We generated a 3D culture of cells in gels in paper by spotting cells suspended in cold (4°C) liquid Matrigel onto the paper and allowing it to gel inside the paper (at 37°C). To ensure that the vertical distribution of cells in gel modules inside the paper was uniform (i.e., cells did not accumulate on the side of the paper on which they were spotted), we surveyed multiple types of commercially available paper. We spotted cells on one face of the paper, and examined the density of cells on both faces of the paper using a confocal fluorescent gel scanner (Figure S1). We found Whatman filter paper #114 (thickness 190 µm, porosity 25 µm) to be the most suitable paper for our cell-culture experiments because: (i) The number of cells on both sides of this type of paper was similar. (ii) This paper did not deteriorate even after several weeks of rocking in culture media (it contains a small amount of proprietary polymer that strengthens the paper). We used this type of paper for all subsequent assays.

#### Confinement of liquids in paper to create in 96-zone array

**Figure 1:**
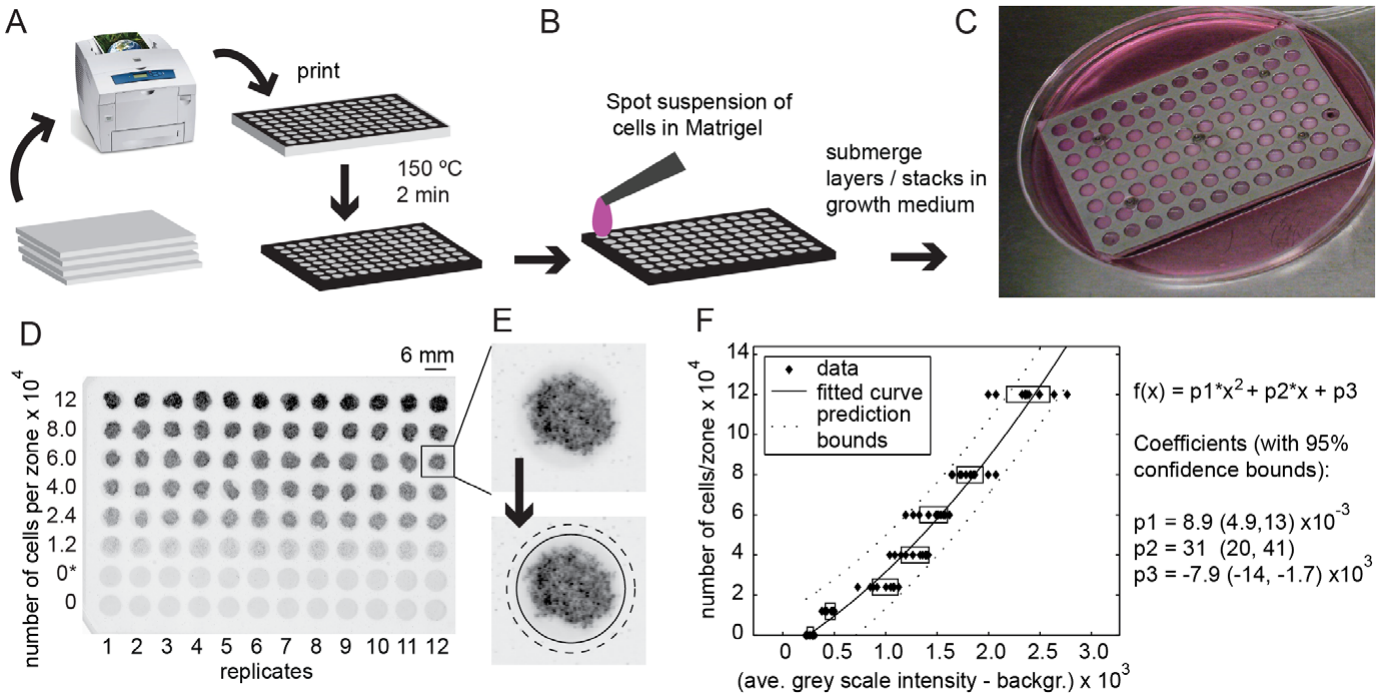
Generation and analysis of multi-zone cultures. (A) Multi-zone plates are printed using commercially-available printer equipped with a solid-ink cartridge. Heating the paper causes the ink on the surface of paper to diffuse into the paper, and to form hydrophobic barriers that separate 96 hydrophilic zones. (B) Cells are seeded into the hydrophilic zones using a parallel or repeater pipette. (C) Stacking 96-zone paper plates assembles 96 3D cell cultures; a metal holder holds compresses them into contact. The 3D cultures are maintained submerged in a common culture medium. (D) We imaged the layers using a fluorescent gel scanner. Image of the 96-zones plate that contained eight concentrations of cells. Black color is proportional to the fluorescence of GFP measured by a gel scanner. (E) Image analysis software mapped the location of each zone, measured average value for grey scale intensity inside each zone (inside black circle), and subtracted the average grey scale intensity outsize the zones (along the dotted circumference). (F) Graph showing correlation between average grey scale intensity and the number of cells for each zone in (D). The fitted curve was used as a calibration curve, which converts intensity of GFP to the number of cells.

The size of the spots that contains cells in gels in paper is defined by the volume of the spotted solution [28]; but the exact shape and position of these spots can be influenced by non-uniformities in capillary wicking. Patterning paper with hydrophobic barriers makes it possible to control capillary wicking of aqueous solutions, since fluid permeated the hydrophilic areas only [35], [36]. The use of hydrophobic barriers also makes it possible to tolerate positional errors in spotting: a drop of liquid spotted anywhere on the hydrophilic area surrounded by a hydrophobic barrier fills the entire hydrophilic area [37]. There are several materials that can be used to produce hydrophobic barriers in paper. There is, however, typically a tradeoff between the convenience of depositing the material in the paper and the impermeability of the barrier produced. Photoresist (SU-8) [35] and polydimethylsiloxane (PDMS) [36] completely block the lateral spreading of water and water-soluble materials. Both patterning methods, however, are time-consuming and therefore not amendable to the demands of high-throughput production. To generate hydrophobic barriers inside the paper, we used a strategy based on solid wax printing [38], because it is simple, fast, and inexpensive. Although we noticed that the wax is somewhat permeable to small molecules and oxygen (see below), it effectively prevents the spreading of solutions and confines the gels and cells within their zones (Fig. 1B). Patterns were generated in two simple steps: i) printing the 96-zone pattern with a commercially available solid-wax printer (Xerox Phaser 8560) into the surface of the paper; ii) heating the paper for 2 min to 150°C to melt the wax and to distribute it across the full thickness of the paper (Fig. 1A) [38].

Although it is possible to generate many 2D patterns using this approach, here we focus on a design used in commercial 96-well plates. The use of this layout facilitates the delivery of cells and reagents onto the sheets of paper using standard multi-channel pipettes and liquid handlers, and their analysis using plate readers, scanners and commercial software.

#### Choice of cell-delivery procedures

At less than 100% humidity and room temperature, several microliters of water spotted on paper evaporate within one minute. To prevent osmotic damage to cells due to evaporation of the Matrigel solution, spotting had to be completed within one minute and the spotted paper should be immersed in media. Alternatively, but less conveniently, the layers could be maintained in a humid atmosphere, or handled partially submerged in media.

We compared the delivery of a suspension of cells in Matrigel onto patterned paper using multi-channel pipette and repeater pipette. Cell plating using repeated spotting was faster than manual alignment of multiple channels with the zones of the multi-zone plate. Spotting, in principle, could also be automated using a standard liquid-handling robot. Spotting of cells suspended in 4°C Matrigel on the hydrophilic regions of the 96-zone plate yielded arrays of gels in the approximate form of cylinders having ∼200 µm thickness and 6-mm diameter (Fig. 1D). The thickness was approximately that of paper.

We observed that the radius of the areas that contained cells was 10–20% smaller than the radius of the zone defined by wax barrier (Fig. 1E). This cell-free rim of Matrigel was produced because the rate of spreading of 5-µm cells inside the paper is slower than the rate of capillary wicking of Matrigel solution. The 20-µm pores in the paper hinder permeation of the cells. The size of the rim increased with increasing time required for Matrigel solution to reach the edge of the pattern. Supplying hydrophilic zone with ∼5-fold excess of volume of suspension of cells in Matrigel minimizes the time liquid travels laterally inside the paper and, thus, alleviates the cell-free rim. Because this method requires five-fold excess of cells and Matrigel, we used it only in some applications (e.g. see below).

### Analysis of multi-zone arrays that contain cells

#### Quantification of cells in a single layer of multi-zone plate

Figure 1D shows an example of an image of a single layer of a 96-zone plate that contained an array of gels that had different concentrations of cells. We generated this array by spotting 4 µL of Matrigel containing different amounts of MDA-MB-231 cells stably transfected with GFP (from 0.1×10^7^ cells/mL to 5×10^7^ cells/mL). Three hours after plating the cells, we imaged the sample using a fluorescence scanner. The average intensity of black color in the image was proportional to the intensity of GFP fluorescence in the sample. We developed an automatic image-processing procedure in Matlab to quantify images of arrays obtained from a gel scanner. Analysis of images of arrays on wet paper was more challenging than analysis of standard rigid arrays (e.g. DNA arrays). Wet papers are soft, and as a result, they tend to compress, bend or buckle when laid on a scanner; array elements in the acquired images from the scanner cannot be mapped easily using a standard grid, and the images cannot be corrected using simple Euclidian transformations (e.g. rotation).

The image-processing procedure we developed allowed the identification of zones of cells while accounting for small, non-uniform distortions (buckle or twist) in the paper (Fig. S6). The basic algorithm for the image analysis software is included as a part of the supporting material. Once the software mapped the coordinates of all zones, it measured the average value of grey-scale intensity inside each zone (inside black circle in Fig. 1E) and subtracted the average grey-scale intensity outsize the zones (along the circumference of the dotted circle in Fig. 1E). The GFP intensity correlated with the number of cells inside each zone (Fig. 1F) and this relationship was used to convert GFP intensity to the number of cells in all experiments.

### Culture and analysis of cells in multiple layers of 96 zones

#### Construction and analysis of multiple 3D constructs with different initial cell distributions

**Figure 2:**
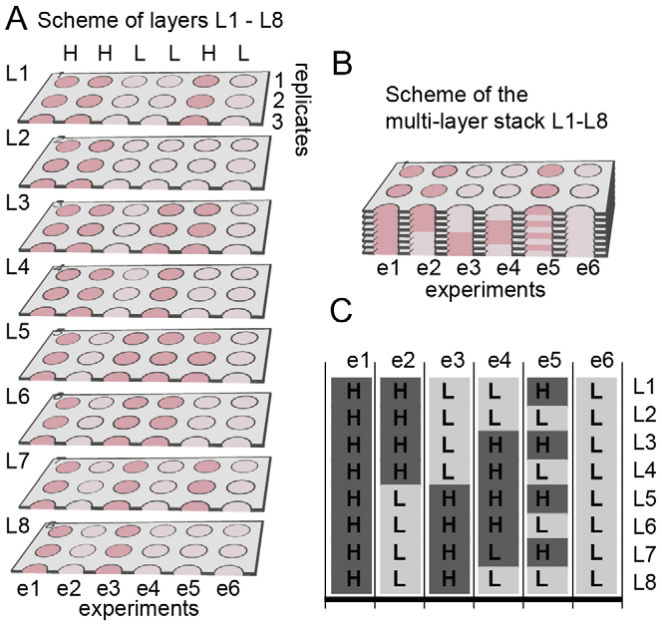
Scheme of the multi-zone, muli-layer sample containing high (H) and low (L) concentrations of cells in specific locations (red, H: 120,000 cells/zone or pink, L: 12,000 cells/zone). (A) Layers prior to stacking. Only three out of eight replicates are shown; to simplify visualizations. (B) Stacking the layers L1 through L8 generates L1L8-stack. (C) Distribution of GFP-MDA-MB-231 cells in layers L1 through L8. Dark grey color denotes zones that contain 120,000 cell/zone (“H”); light grey color denotes those that contain 12,000 cells/zone (“L”). Experiments are separated by vertical black lines.

Stacking 200-µm-thick multi-zone arrays that contained gel slabs with cells (Fig. 2A) created arrays of multi-layer cultures (Fig. 2B). Each multi-layer culture within this array was composed of 200 µm-thick slabs of gel supported by cellulose fibers; each slab can contain different cell types or different concentrations of cells (Fig. 2A–C). Multi-zone multi-layer culture, thus, allows the assembly of arrays of 3D cultures of many compositions at once. Controlling the composition of the constructs makes it possible to manipulate gradients of oxygen and nutrients in these constructs, and to trace the migration of cells inside them. All would be challenging in standard 3D culture techniques (e.g., culture of cells in 3D gels, or 3D aggregates of cells), because controlling spatial distribution of cells in these constructs is not practical.

Using these capabilities, we characterized growth and migration of breast-cancer cells in 3D constructs. We generated eight layers that contained different concentrations of MDA-MB-231-GFP breast cancer cells in different zones of a multi-zone plate (Fig. 2C; Fig. S2A). Specifically, we spotted 4 µL of low (L, 3×10^6^ cells/mL) and high (H, 3×10^7^ cells/mL) densities of these cells in Matrigel onto the multi-zone arrays. For convenience, we refer to zones that contain these concentrations as “L-zones” and “H-zones” respectively. Stacking eight arrays that contained different distributions of H and L zones generated 48 sets of 1600-µm-thick cultures (six different geometries, e1–e6; eight replicates for each); each geometry contained a defined vertical distribution of H and L zones (Fig. 2B–C). For example, stacking eight H zones created a vertical sequence we refer to as HHHHHHHH; collectively, this stack corresponds to a 1600-µm thick 3D-culture that contains a 120,000×8 = 960,000 cells distributed uniformly (Fig. 2C, experiment e1). We stacked these layers on top of a sheet of cellulose acetate. This polymer is impermeable to water and oxygen; nutrient and oxygen diffused into the multi-layer culture only from one end (through the fore of the layers in contact with medium: i.e., L1). Cells in the layer L8 had the least access to these factors.

#### Distribution of cells over time

**Figure 3:**
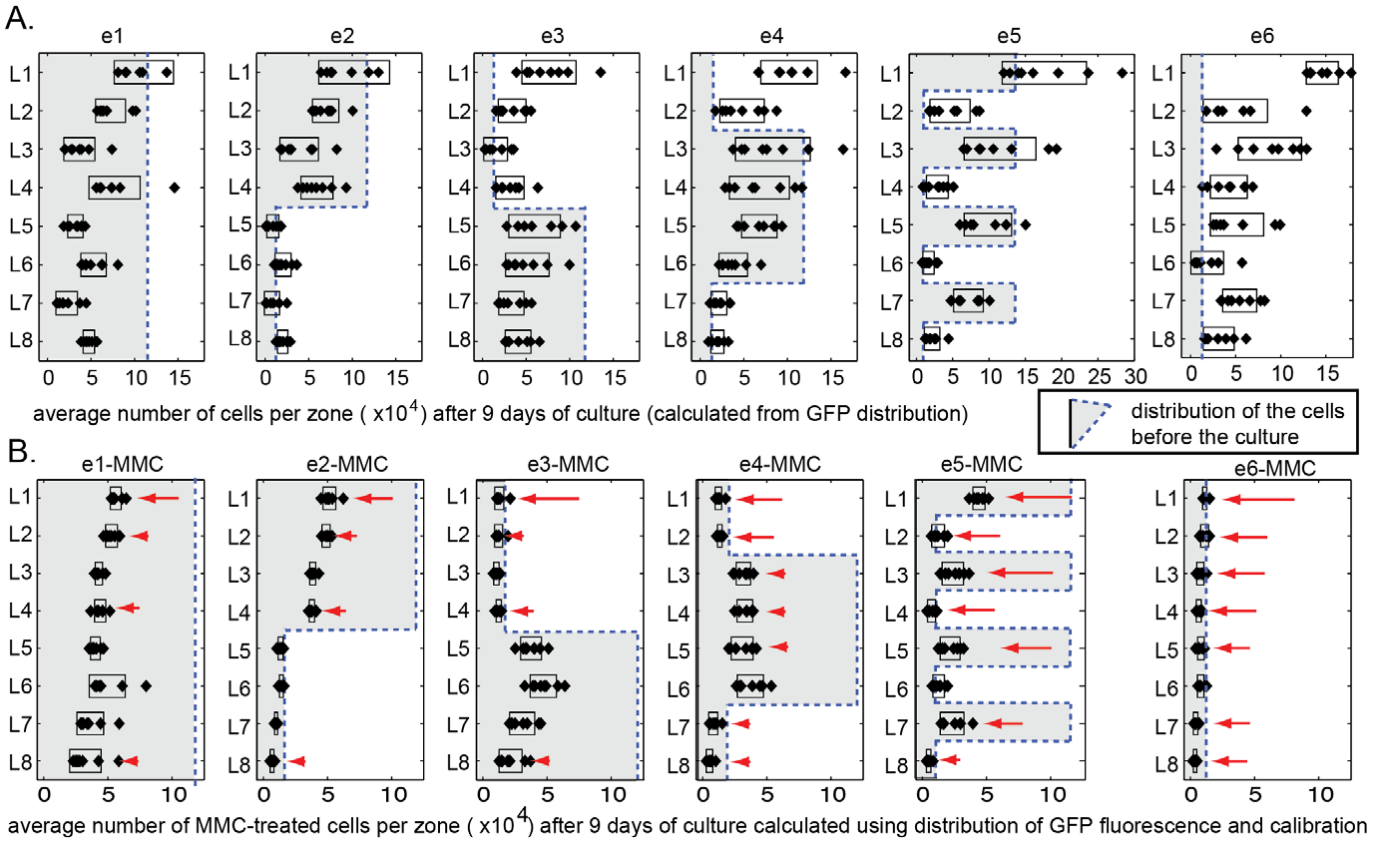
Distribution of cells in multi-zone multi-layer samples described in Fig. 2 before and after nine days of culture. (A) Average concentrations of cells per zone were calculated from average intensity of the GFP in the zone using curve described in Fig. 1F. The grey outlined area depicts the initial number of cells in each sample. (B) We assembled the cultures identical to those described in Fig. 2C using GFP-MDA-MB-231 cells arrested with MMC. The graph represents the average number of cells after nine days of culture. The graphs present data from eight replicates; the width of the overlaying bar is 2x (standard deviation). In the locations marked by red arrows, the number of cells in the presence of MMC was significantly lower (p<0.05) than that in the absence of MMC.

After nine days of culture, we de-stacked the multi-zone arrays by peeling the layers apart, imaged the intensity of GFP in each zone (Fig. S2B), and calculated the total number of cells in each zone of each layer using a calibration curve. We compared the distribution of cells after nine days of culture (Fig. 3A) with the initial number of cells in each layer (grey outline in Fig. 3A). Although the half-life of GFP in live cells can be up to two days [39], the presence and absence of emission due to GFP correlates with the presence of live and dead cells in 2D assays [40]. To confirm the same correlation in our 3D assay, we stained the samples with calcein to visualize metabolically active cells, and with propidium iodide (PI) to visualize necrotic cells with compromised membranes. The distribution of calcein stain coincided with the distribution of GFP fluorescence (Fig. S2). GFP fluorescence in the areas occupied by PI(+)/calcein(-) cells was low. The total number of cells per layer decreased progressively with increasing distance from the oxygenated medium (Fig. 3A). Distributions of cells were similar to those observed in our previous studies [28]. They are expected because oxygen and nutrients diffuse primarily vertically from L1 to L8. The consumption of oxygen and nutrients by cells in the top layers (L1 to L4) depleted them for cells in L5–L8.

The increase or decrease in the number of cells over nine days of culture depended on the initial density of seeding, as well as the relative location of cells inside the 3D cultures.

For example, in cultures presenting uniformly high densities of cells (HHHHHHH), the number of cells decreased in every layer (Fig. 3A, e1), while in a LLLLLLL-stack, the number of cells increased in every layer (Fig. 3A, e6). These changes might have occurred due to cell proliferation, cell death, and/or cell migration from layer to layer. To assess the role of cell division, cell death, and cell migration separately, we assembled stacks in which the distribution of cells were identical to those in Fig. 2. We changed, however, three variables in order to examine the origin of the distribution of cells in the 3D culture: (i) We assembled the stacks using MDA-MB-231-GFP cells treated with Mitomycin C (MMC). This compound arrests division of cells and allows decoupling of proliferation of cells from cell death or migration. (ii) We assembled the stacks from MDA-MB-231 cells that were transfected with GFP or mTomato. Placing cells labeled with different fluorescent markers in specific locations allowed quantification of the migration of cells between the layers. (iii) Combining (i) and (ii) allowed quantification of cell migration independent of cell division.

#### The effect of MMC

By comparing the number of cells in MMC-treated and untreated samples in specific locations, we could infer whether cells were dividing in these locations. In the locations marked by red arrows in Fig. 3B, the number of cells in the presence of MMC was significantly lower (p<0.05, t-test) than that in the absence of MMC. Since MMC stops cell division, the observed decrease in cell number in the presence of MMC suggested cell division must have occurred at the same locations in untreated samples. Cell division was obvious in some layers as an increase in the total number of cells (e.g. layers in e6). As expected, MMC-treatment led to a decrease in the number of cells in those locations.

In other locations, the cell number did not change compared with the initially plated number of cells (e.g. layers L1, L5 and L6 in experiment e2, Fig. 3). Comparing MMC-treated and untreated samples indicated that a constant number of cells in L1 was maintained, we presume, due to a balance of cell division and cell death. Addition of MMC disrupted this balance in L1 and led to a decrease in the number of cells (L1, e2 vs. e2-MMC, Fig. 3). Addition of MMC did not change the number of cells in L5 nor L6 (L5 and L6, e2 vs. e2-MMC, Fig. 3). No cell death, or cell growth occurred in these locations over the period of nine days.

This analysis was based on the assumption that the number of cells that migrate between the layers is low. We confirm this assumption in the experiments below.

#### Analysis of migration of cells

**Figure 4:**
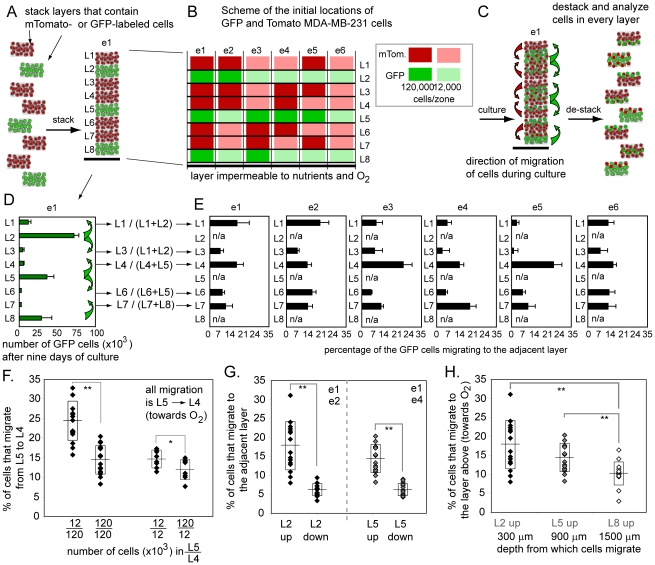
Scheme and the results of the migration experiment. (A) 3D culture composed of the layers that contain MDA-MB-231-GFP-cells in layers L2, L5, L8 and MDA-MB-231-mTomato cells in the other layers. (B) We created multi-layers culture identical to that described in Fig. 2 using GFP- and mTomato-labeled cells. Zones marked by green and red color depict location of Tomato and GFP cells respectively; intensity of color depicts concentration (C) Migration of GFP cells can be detected as increase in GFP fluorescence in layers L1, L3, L4, L5, L6. (D) After nine days of culture, we destacked the layers, quantified GFP fluorescence in each zone of each layer (experiment e1 is used as example). GFP cells are present in layers L1, L3, L4, L5, and L6 due to the migration from layers L2, L5, and L8. (D) Graph describing the percentage of GFP(+) cells that migrated. (F) Migration depended on the relative number of cells in “sender” and “receiver” layers. (G) For layers that contained similar number of cells, migration of cells was directional: significantly more cells migrated to the upper layer (towards oxygen) than to the lower layer. (H) Migration of cells to the upper layer depended on the location of the cells inside the stacks: cells in hypoxic layer L8 migrated significantly less that those in layers L1 and L5 with higher oxygen concentration.

To track the migration of cells in 3D, we generated 3D cultures in which the distributions of cells in space were the same as those in Fig. 2 and 3 using MDA-MB-231 cells stably transfected with GFP and mTomato. Layers L2, L5 and L8 contained GFP cells and layers L1, L3, L4, L6, L7 contained mTomato cells (Fig. 4A). We analyzed the distribution of GFP and mTomato cells after nine days of culture (Fig. 4B). Cells migrated to the adjacent layers (Fig. 4C–D), but the number of cells that migrated was <20% of the original amount of cells seeded in the layers (Fig. 4E). The changes in the distribution of cells due to migration (a factor of <1.2) were significantly less than those caused by local proliferation, or by death of cells (factors of 2–3).

There were many interesting patterns in migration. The number of cells that invaded the adjacent layers depended on several parameters: i) the concentration of cells in the original (“sender”) zone and adjacent (“receiver”) zones; ii) the position of the “sender”-zone inside the stack, and iii) the order of the “sender” and “receiver” zones (i.e. the directionality of migration).

We calculated the fraction of GFP cells that migrated as a ratio of GFP fluorescence in the “receiver”-zone to that in the “sender” zone at the end of the 9-day culture (e.g. for migration from L2 to L1, the fraction was defined as GFP(L1)/(GFP(L1)+GFP(L2)) at day nine). This fraction was the highest (up to 20%) when the “sender”-zones contained high densities of cells (e.g., number of cells plated on day 0 was 120,000 cells/zone) and the “receiver” contained a low number of cells (12,000 cells/zone) (Fig. 4F). The fraction of cells that migrated between the layers that contained similar numbers of cells (e.g., both “sender” and “receiver zone contained 120,000 cells/zone) was also significant (5–15%, depending on the location).

We further observed that migration was directional: 2–3 times more cells migrated toward the layer above, i.e. (towards a higher concentration of oxygen and nutrients) than toward the layer below (Fig. 4G). The fraction of cells migrating toward oxygen and nutrients depended on the original positions of the cells inside the 3D stack: migration of cells in hypoxic layer L8 was 50% of that in layers L1–L5 (Fig. 4H). This observation is compatible with migration of cells toward high O_2_ levels along the oxygen gradient of oxygen established inside the stack. We hypothesize that the magnitude of this gradient is the largest in layers L1–L5. Examination of the rate of proliferation (Fig. 3) suggested that layers below L6 have equally low concentrations of oxygen. The magnitude of the oxygen gradient (∂[O_2_]/∂z) in these layers was nearly zero, since the concentration of O_2_ was uniformly small within them.

To check whether the presence of the fluorescent reporter in the cells had any effect on the final distribution of cells, we used a control sample in which the locations of cells were reversed (mTomato in L2, L5, L8, and GFP in L1, L3, L4, L6, L7). Trends for the “reversed” experiment were similar (Fig. S3).

#### The effect of cell proliferation on migration of cells

To determine whether migration of cells to an adjacent layer could occur in the absence of proliferation, we analyzed cell migration in stacks assembled from GFP and mTomato MDA-MB-231 cells arrested with MMC. We used these cells to generate 3D cultures in which the distributions of cells in space were the same as those in Fig. 4. We observed that MMC-arrested cells migrated to the adjacent zones (Fig. S6A). Although the number of migrating cells was lower than that in cultures containing proliferating cells, the fraction of migrating cells was similar (<25%). The pattern of cell migration in 3D cultures containing growth-arrested cells (Fig. S4) was similar to that in the cultures containing non-arrested cells (Fig. 4). The fraction of migrating cells correlated with the direction and magnitude of the gradient of oxygen (Fig. S4D-E). The fraction of growth-arrested cells migrating towards oxygenated medium was higher than the fraction of cells migrating away from it. Similarity in migration patterns of MDA-MB-231 cells and identical cells arrested with MMC suggested that migration of these cells in 3D hydrogels was independent of cell division.

#### Examination of growth and migration of cells using 3D spatial distributions of cells

**Figure 5:**
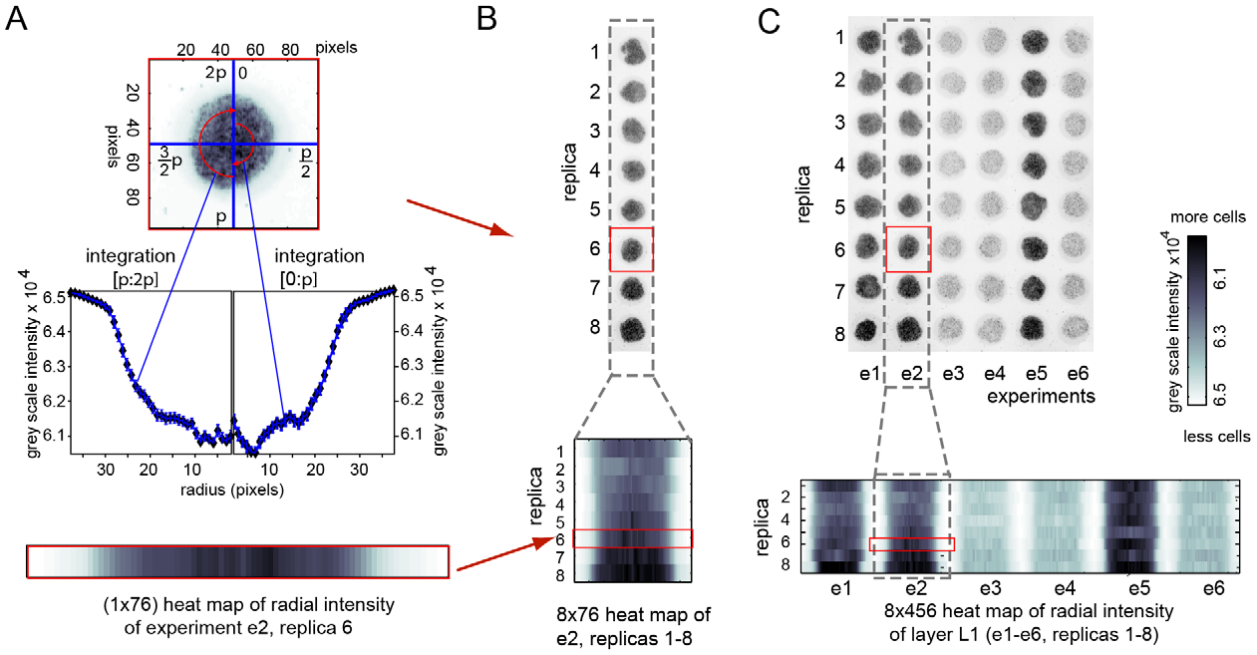
Heat map representation of the radial distribution of the fluorescent intensity in each zone of the multi-zone plate. (A) We defined polar coordinate system (*r*, *ϕ*) for each zone (*r* = 0 in the middle of the zone, *ϕ* = 0 on the axis dividing the zone in half). Integration of the image in *ϕ*  = (0; π) and *ϕ*  = (π; 2π) range for discrete *r* yielded left and right radial distribution of gray scale intensity. For a zone with a radius of 3.0 mm imaged with 100 um resolution, we measured 33 radial distributions for r = 1,2,...,33 pixels (the outer 10% of the distribution represent an intensity of background fluorescence). Both radial distributions can be presented in a 1×76 heat map. (B) We “stacked” radial distributions from zones in the same column that correspond to the replicates of the same experiment; here, eight replicates are visualized as 8×76 heat map. (C) A sheet that contains six experiments with eight replicates each can be presented as 8×456 heat map. In this example, we prepared two suspensions of MDA-MB-231-GFP cells in Matrigel (high, 3×10^7^ cells/mL and low concentration, 3×10^6^ cells/mL) and spotted 4 µL of these suspensions on zones of a 48-zone plate. The image was acquired using fluorescent scanner and the intensity of black color is proportional to GFP fluorescence; heat map, hence, also represents distribution of fluorescence of GFP in this sample.

The preceding analyses were based on average distributions of cells in each zone. Because we imaged each layer with a gel scanner at 100-µm (lateral) resolution (Fig. S2), it was also possible to reconstruct the distribution of cells in 3D with 100–200 µm resolution. Representing 3D distributions is challenging, but 3D cell constructs of cylindrical shape have rotational symmetry. A 2D radial distribution of fluorescent intensity can represent the distribution of cells in these constructs. Distribution of cells in these 3D cultures might, in principle, deviate from central-symmetry. To check the symmetry of the distribution, we calculated half-radial distributions and plotted them side-by-side in all analyses. In all our experiments, the distributions were close to central-symmetric and the two half-radial distributions were very similar (Fig. 5); they were not similar, however, if the left and the right halves of the 3D construct were not identical (see below).

**Figure 6:**
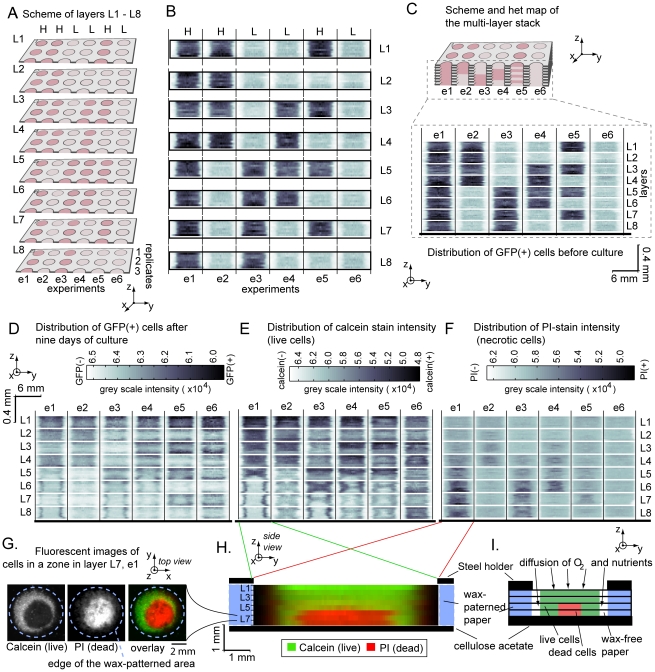
Heat map representation of the radial distribution of intensities in multi-layer experiments. (A) Scheme of the multi-zone sample described in Fig. 2 (red: 120,000 cells/zone or pink: 12,000 cells/zone). Only three out of eight replicates are shown; to simplify visualizations. (B) Heat map representation of the radial distribution of GFP fluorescence of layers L1 through L8; experiments are separated by vertical black lines (see Fig. 5 for details of heat map). (C) Stacking the layers L1 through L8 generates L1L8-stack. Stacking eight heat maps generates a heat map which describes the whole L1L8-stack. Conveniently, coarse view of the map provides an estimate of fluorescent intensity at the cross-section of the L1L8 stack, whereas the fine structure of map provides an estimate of variation of intensities within each zone or variability between different zones. (D–F) Nine days after stacking and culture, we de-stacked the layers and quantified the distribution of GFP fluorescence (D), distribution of live cells that stain with calcein (E), and distribution of dead cells that stain with propidium iodide (PI) (F). (G) Images of stains in each zone demonstrate that lateral distributions of intensity of calcein and PI inside the zones are complimentary. Image in (H) is an overlay of rescaled heat maps from (E) and (F); the image demonstrates that viable cells reside in the shell of 6 mm in diameter and ∼800 micron in thickness. (I) Scheme describing possible path of diffusion of oxygen and nutrients through the stacks.

To calculate half-radial distributions, the image analysis script divided a circular zone into multiple concentric rings (the width of each ring is 1 pixel). Calculating the average intensity of the pixels in each of the two halves of the ring yielded two scatter plots describing the radial distribution of grey-scale intensities in the zone (Fig. 5A). We plotted two half-radial distributions of cells in each zone as a heat map (color coded 2D array) (Fig. 5A, bottom), and we grouped the heat maps from multiple zones such that replicates of the same condition are stacked on top of each other (Fig. 5B). Fig. 5C shows a heat-map representation of the 48-zone plate that contains six columns and eight rows. Each column is a separate experiment and each row is a replicate of the experiments in the same column. Fig. 6A–C describe how heat maps calculated for individual layers can be stacked to visualize the concentration of cells in multi-zone, multi-layer stacks. Specifically, the heat map in Fig. 6C describes the initial 3D distribution of cells in the eight-layer-stack described in Fig. 2.

#### Quantification of spatial distribution of cells in multi-layer stacks

We analyzed the distribution of GFP intensity in every layer after nine days of culture, and generated stacks of heat maps using our custom computer software. Comparison of the 3D distribution of cells after the nine-day culture period revealed that the changes in numbers of cells for a given layer is dependent on the position of the layer within the stack and the total number of cells in the layers above it. (Fig. 6D). The distribution of live and dead cells was not uniform in L5–L8: dead cells were found close to the center of the zones whereas cells survived close to the rim of the zones (Fig. 6E–G). The width of this “rim of survival” was 400–800 µm (Fig. 6H). This observation suggested that the nutrients and oxygen can access the multi-layer culture through the cell-free rim that is between the cell-containing area and the wax-patterned area (e.g. Fig. 6G–I). Diffusion of oxygen and nutrients through this 1 mm rim could to be sufficient to supply cells in layers L5–L8 (Fig. 6I). The flux of O_2_ and nutrients could also occur through the wax borders.

#### Changing the diffusion profile of oxygen and nutrient around the 3D cultures

**Figure 7:**
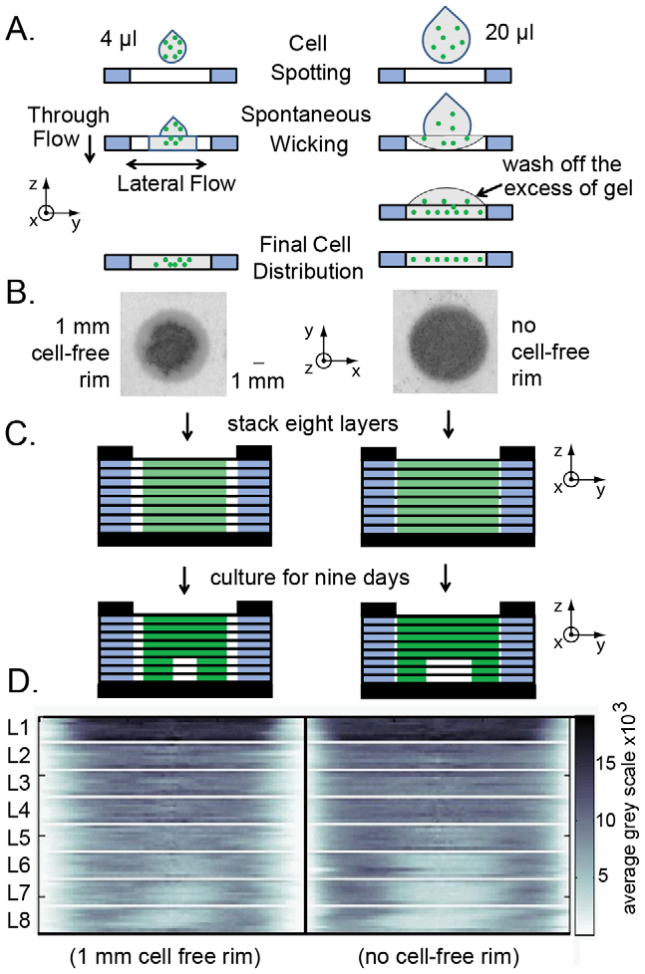
Controlling lateral distribution of cells in 3D stacks. (A) Schematic describing the effect of spotting volume on final distribution of cells in a zone (B) Distribution of GFP intensity (proportional to black color) in zones spotted with 4 µl and 20 µl of suspension of MDA-MB-231-GFP cells in Matrigel. (C) We stacked eight sheets that contained cells in gels depicted in (B), and analyzed distribution of cells after nine days of culture. (D) Heat map representation of the radial distribution of GFP fluorescence of layer L1 through L8; experiments are separated by vertical black lines, layers are separated by white lines (see Fig. 5 for details of heat map).

**Figure 8:**
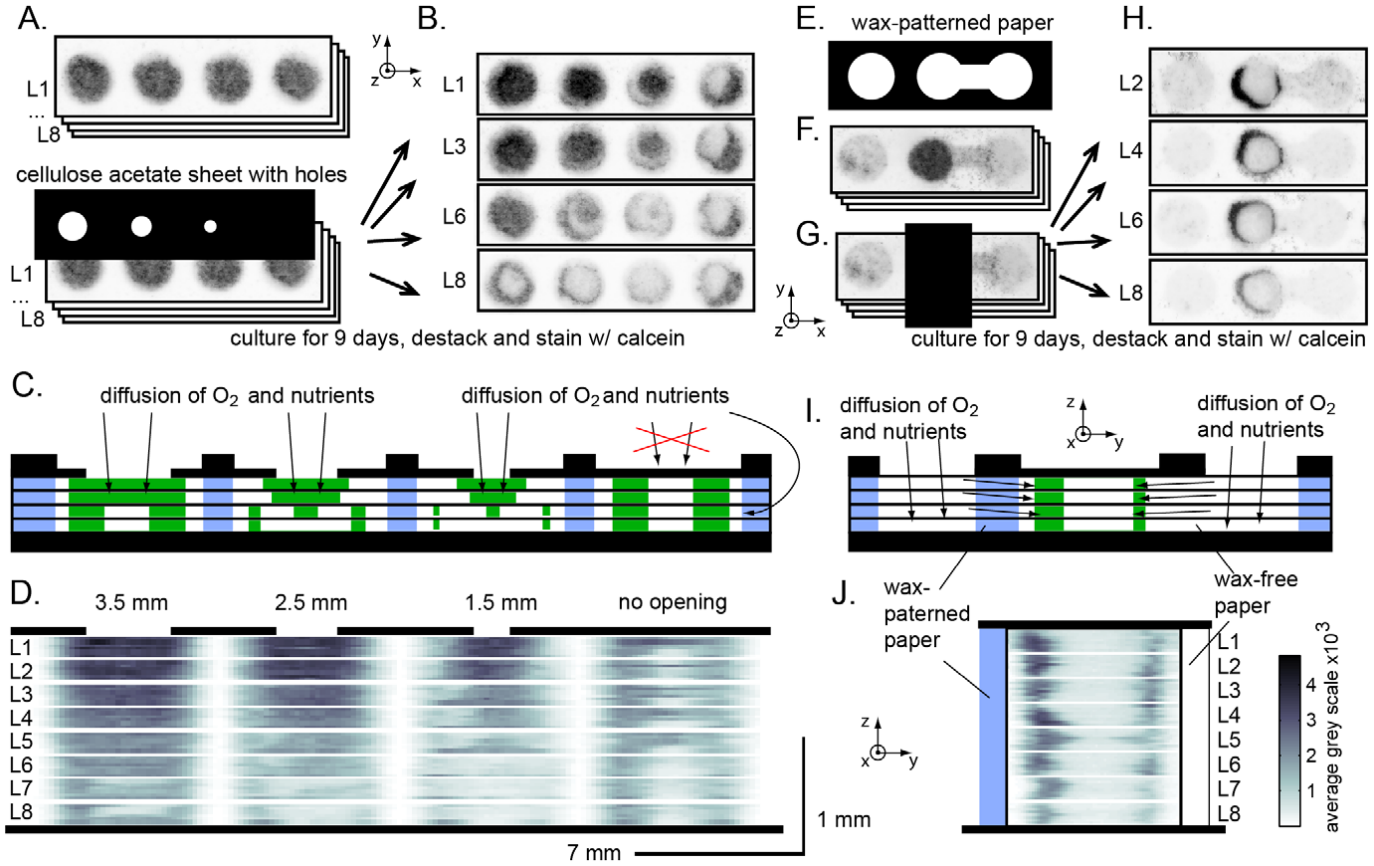
Effect of diffusion barriers on cell distribution. (A) Scheme depicting use of a cellulose acetate sheet with holes to control the diffusion of oxygen and nutrient to the cells in 3D gels from the bulk media. All layers (L1–L8) contained uniform concentrations of cells in all zones (40,000 cell/zone in Matrigel). (B) Representative images of the zone layers after nine days of culture; the samples were stained with calcein. (C) Scheme of the distribution of cells (green) in stacks and source for diffusion of oxygen/nutrient through the opening in the cellulose acetate layer atop of each stack (black) or through the wax-patterned paper (blue). (D) Heat map of the radial distribution of calcein stain in the stacks depicted in (C); the size of the holes are drawn to scale. (E–H) Experiments that provide a qualitative comparison of the diffusion rates of oxygen/nutrients through wax-printed (left side) and wax-free paper (right side). (F) Cells were spotted in the middle circle of the wax-printed pattern (E). We stacked eight layers depicted in (F), covered the cell containing zone with cellulose acetate and cultured the construct for nine days. (H) After de-stacking the layers, staining live cells with calcein and scanning the samples demonstrated that more cells resided next to the wax-patterned area than next to the wax-free area. (I) Scheme describing side view of the stack and heat map (J) describing radial distribution of calcein intensity in the stack. Images in (A–H) were acquired using fluorescent gel scanner (100 µm resolution), black color is proportional to the intensity of green fluorescence (calcein viability stain). See Fig. 5 for details of heat map in (D) and (J).

In order to determine the primary source of diffusion of oxygen and nutrients to the cells in the rim of the layers L5–L8, we modulated the diffusion profile through the layers using two approaches: (i) Increasing the area occupied by cells within each zone decreased the size of cell-free rim through which diffusion of oxygen and nutrient occurred from the bulk media (Fig. 7). (ii) Placing a perforated sheet of cellulose acetate on top of L1 controlled the size of the opening through which diffusion of oxygen and nutrient occurred (Fig. 8).

#### Diffusion of oxygen and nutrients does not occur through the cell-free rim

To test the first approach, we spotted cells in each zone using an excess of a suspension of cells in Matrigel, and eliminated the cell-free rim in the zone (Fig. 7A). Spotting a zone that has a volume of 4 µL with 20 µL of suspension of cells in Matrigel rapidly covered the whole zone with excess of liquid. Using a 5x-excess of cell suspension (20 µL) minimized the lateral flow of liquid inside the paper and decreased the size of the “cell-free rim”. Cells trapped in Matrigel in paper were distributed uniformly throughout the zone (Fig. 7B); the excess was washed off 1–3 minutes after spotting. To see the effect a cell-free rim has on the 3D distribution of viable cells in an eight-layer stack, we seeded individual zones with ca. 40,000 cells (using two approaches described in Fig. 7A), stacked the layers, and cultured the stacks for nine days. Fig. 7B shows that the elimination of the cell-free rim did not change the overall 3D distribution of cells. Similar or even higher number of viable cells accumulated on the outer rim of zones in layers L5–L8. These observations suggested that diffusion of oxygen and nutrients might occur through the wax-patterned areas around the cell-containing areas.

#### Diffusion of oxygen and nutrients occurs through the wax-patterned paper

To eliminate any possibility for diffusion of oxygen/nutrient through the top rim, we overlaid the cultures with a layer of cellulose acetate that contains a hole with diameter significantly smaller than the diameter of the cell-containing zone. We covered a stack of eight multi-zone arrays of cells in Matrigel (7-mm zones, 40,000 cells/zone) with cellulose acetate sheet that contained an arrays holes of 3.5, 2.5, 1.5 mm in diameter (Fig. 8A). The centers of the holes were aligned with the centers of each cell-containing zone. Some zones were covered with a continuous sheet of cellulose acetate (“no opening”, Fig. 8C). After nine days of culture, we stained the individual layers with calcein and analyzed the 3D distribution of live cells. Decreasing the diameter of the opening decreased the cell viability in layers L5–L8, and changed the overall 3D distribution of viable cells (Fig. 8C, D). Nevertheless, we consistently detected live cells on the outer rim of the cylindrical 3D cultures using opening of any size, including those that were covered by cellulose acetate without any opening. These observations confirmed that permeation of oxygen and some other nutrients occurs through wax-patterned paper.

#### Oxygen diffuses faster through the wax-patterned paper than through non-patterned paper

We compared the permeability to oxygen/nutrients of paper permeated by wax and non-patterned paper (permeated by water when submerged in medium). Printing the asymmetric pattern depicted in Fig. 8E made it possible to compare the oxygen/nutrients to the cell-containing zones through non-patterned paper (from the right) and wax-patterned paper (from the left) (Fig. 8E). Plating the cells in central zone and stacking eight identical layers generated a cylinder of cells in Matrigel that contained a stack of wet paper on the right side and wax-patterned paper from the opposite side (Fig. 8F). We closed the top of the culture with cellulose acetate (Fig. 8G), cultured for nine days submerged in growth medium, stained the individual layers with calcein, and imaged distribution of live cells by gel scanner (Fig. 8H displays representative images from some layers).

Analysis of half-radial distributions in left and right half of the zones (Fig. 8I–J) demonstrated that significantly higher number of cells resided along the rim of the zones adjacent to the wax-patterned paper; fewer cells resided on the side adjacent to non-patterned paper. No live cells were present in the middle of the cylinder. These experiments demonstrated that: (i) Diffusion profiles in the arrays of 3D gels can be changed using a variety of approaches (e.g. by changing of the spatial composition and shape of cultures or by changing the permeability of the environment surrounding the cultures). (ii) Paper permeated by wax facilitates high-throughput plating of cells and resists initial spreading of aqueous solvents. It is, however, permeable to oxygen and solutes after prolonged incubation in culture medium. We are currently investigating other strategies for patterning of paper that can create barriers impermeable to oxygen.

## Discussion

### Paper-supported multi-layer cultures vs. multicellular spheroids

Multi-layer, multi-zone cell culture platform made it possible to observe how the long-term proliferation and migration of live cells in 3D tissues were influenced by initial cell density, effective diffusive area, and other parameters that could be regulated in our multi-layer 3D construct. This level of control over the distribution of cells and gradients in 3D is lacking in conventional 3D culture systems. As an example, we compare, side-by-side, the advantages of our system over that based on cellular spheroids (3D cellular aggregates), which are common models of solid tumors used in high-throughput screening applications: (1) the spatial composition of spheroids is very difficult to control. Their geometry is limited to spherical aggregates of different size, uniformly filled with cells of one type. In contrast, stacking multiple cell-containing sheets assembles a structure similar to a spheroid (Fig. 6H). Specific cell types (or cell concentrations) can be easily placed in specific locations (e.g. Fig. 4). (2) Spheroids are inherently symmetric, they can only contain central-symmetric gradients (and distributions of cells). In contrast, we have demonstrated that the incorporation of diffusion barriers in multi-layer cultures creates gradients of complex shapes in 3D (Fig. 8). (3) The ability to de-stack the 3D construct bypasses several days of preparations required for physical sectioning. Unlike microtome sectioning, peeling the layers apart yields viable cells that can be further cultured, re-stacked or analyzed. (4) There are many reported examples of high-throughput assays in which 3D spheroids are distributed in the wells of 96-well plates [20], [41], [42]. These assays provide information about the average behavior of cells in 3D aggregates. Multi-layer culture, however, makes it possible to collect thousands of data, which collectively describe the distributions of cells in each 3D substrate in detail.

### Layer-by-layer assembly of heterogeneous tissues to study cell migration in 3D

Multi-layer assays enable the analysis of 3D migration of cells between layers of paper that contain different types of cells. This analysis yields significantly more information than that obtained from any standard high-throughput 3D migration assays (e.g. invasion through Matrigel plugs in Transwell™ plates). Cell migration in multi-layer cultures that contained defined 3D distribution of cells is more physiologically relevant than that in conventional migration assays in which cells invade “blank” hydrogel (e.g. Transwell™ migration) or 2D areas free of cells (wound-healing assay). Studying the invasion of cells into cell-free ECM hydrogels is not an accurate model, because in physiological settings, all tissues usually contain a non-zero concentration of cells (e.g. stroma cells) [43], [44]. We have investigated migration in the constructs that contained two variants of the same cell line (GFP- and mTomato-labeled breast- cancer cells). Analogous geometries can be assembled and analyzed using any other cell types (e.g. cancer cells and stromal cells [45]). Cultures that contain more than two distinct cell types (e.g., cancer, stromal, and endothelial cells) can be assembled in the same manner, provided that each cell can be traced using a distinct fluorescent reporter, or cell specific marker (e.g., CD31 for endothelial cells).

There are several techniques for the assembly of 3D cultures of defined spatial composition, in which investigation of migration is possible [46]–[51]. Processing hundreds of assembled 3D aggregates and interfacing them with the current infrastructure for high-throughput screening (multi-well platforms, confocal imagers) can be challenging. The use of arrays of 3D cultures supported by a single sheet dramatically simplifies parallel handling of hundreds of 3D cultures. Grouping 3D cultures on the same sheet also enables parallel sectioning and analysis of large number of 3D cultures with 200-µm resolution. This technique is much faster than standard sectioning tools.

### Multi-zone multi-layer culture platform can be implemented in many formats and materials

Because the substrates are printed on a commercially-available printer, generating substrates that contain an arbitrary number, size, shape and spatial density of cell-containing zones can be performed simply by altering the design in a drawing software. We used these capabilities to change the shape of the wax barrier around the cultures and, thus, change diffusion profile of oxygen and nutrients around the culture (Fig 8E-J). This approach is not limited to paper made of cellulose: Wax printing can be easily used to create hydrophobic barriers in other substrates that are thin, porous and hydrophilic (e.g. silk [52]–[54], poly-lactic-glycolic acid [55], [56]). Depositing cells onto these substrates would create arrays of cells in 3D gels supported by custom porous hydrophilic matrix, which, if necessary, can be biodegradable. Paper can be used with gels that can be triggered to polymerize inside the porous paper matrix (e.g. collagen, ionotropic gels [57], synthetic self-assembling hydrogels [58]–[60], chemically cross-linked gels [61] or photo-cross-linked gels [62]). Additionally, paper can be chemically-modified to present peptides [63], [64] and other bioactive compounds [65], [66].

### The need for high-content data analysis

Simple 2D analysis, which was based on average number of cells in each layer, yielded useful information about directionality of migration, and survival of cells in different regions of the stack (Fig. 3–4). The permeability of wax to oxygen makes analysis of 3D cultures more complex. The shape of gradients is three-dimensional, and only 3D analysis provides a complete description of the distribution of cells (Fig. 6). Although no special equipments was required to collect data for 3D distribution of cells, reconstructing the data from 2D images of each layer *in silico* generated a lot of information-568 (71×8) data points characterized the radial distribution of intensity in each 8-layer stack. Processing of these data requires multivariate statistics, which is more challenging than our 2D analysis (e.g. pair-wise t-test in Fig. 3). We expect that bioinformatic tools that process information from high-content screens will facilitate accurate analysis of 3D distributions in our system [67]–[69].

### Conclusions

We demonstrated that multi-layer cultures allowed the examination of the behavior of cells in 3D cultures of well-defined geometries and composition. We have used a limited number of cells types and studied limited number of cellular responses. There are no limitations, however, to expanding this approach to any cell types that can be cultured inside ECM hydrogels, and any responses that can be measured using fluorescent readout. The simplicity of the plating and stacking steps, and the use of substrates patterned into a standard 96-well format, will enable automation of these steps using standard high-throughput liquid-handling robotics. We believe that the simplicity of the patterning and stacking technology will make it possible for researchers in the biomedical community to use this approach to design custom platforms for high-throughput 3D cultures for specific applications.

## Materials and Methods

### Cell Culture and Transfection

Reagents for cell culture and analysis were purchased from Invitrogen unless otherwise noted. MDA-MB-231 cells (ATCC) were cultured as recommended by ATCC in Eagle's Minimal Essential Medium (EMEM, ATCC) supplemented with 10% fetal bovine serum (FBS), 1% GlutaMax™, and 1% penicillin/streptomycin at 37°C and 5% CO_2_ in a humidified incubator. The cells were transfected by lentivirus (GFP) or retrovirus (mTomato) in the presence of 5 µg/ml polybrene (Sigma) as described in [70]. The cells were expanded and sorted for GFP(+) (∼30%) or mTomato(+) (∼10%) population. The intensity of the label did change for >20 passages. For experiments, we used cells of passage 20 or lower (post-transfection).

### Printing and cutting the multi-zone arrays

Multi-zone designs (available upon request) were drawn in Illustrator CS4 (Adobe) and directly printed to an 8.5″ × 11″ paper substrate by a color wax printer (Phaser 8560DN, Xerox). The wax-printed paper was then baked in an oven (150°C, 2 min) to melt the wax and cause it to penetrated through the entire thickness of the paper [38]. After baking, the paper was submerged in deionized water (di-H_2_O) and after the expansion (ca. 2.2% by width and 0.5% by length) the wet paper was cut using a laser cutter (Versa Laser-Universal Laser VL-300, 50 Watt). We rinsed the cut paper in di-H_2_O (3×1 hour) to remove the burnt paper residue on the edges of the pattern.

### Sterilization of the substrates

Sterilization of the wax-patterned paper was problematic because prolonged heating of patterned substrates above >100°C caused the wax to melt and to spread laterally across the paper blurring the patterns. Immersion of wax-printed paper in water during autoclaving at 120°C blocked the spreading of wax, and the 96-zone pattern was preserved. We believe that this strategy worked because hydrophobic wax could not displace water from the hydrophilic surface of paper. Heating a printed sheet of paper under water rendered the wax barriers hydrophilic, however. This change in hydrophobicity could be explained on the assumption that the wax is amphiphilic: heating the wax in water caused the molecules to rearrange and expose polar groups at the interface with water. We restored the hydrophobicity of the wax barrier heating the dried paper at 120°C for one minute. This treatment redisplayed the hydrophobic groups on the surface and the hydrophobicity was recovered. Simpler sterilization could, in principle, be accomplished using low-temperature sterilization techniques (e.g., treatment with ethylene oxide).

### Plating cells onto the multi-zone arrays

We detached the cells using treatment with trypsin-EDTA (5 min), washed with serum-containing media, and suspended in growth factor-free Matrigel (BD biosciences) at a final concentration of 2×10^7^ cells/mL or other concentrations for calibration curve (Fig. 1). Unless specified otherwise, Matrigel was diluted with cold media 1∶1. Usually, we spotted 4 µL of this suspension onto each zone of the paper using a repeater pipette (Gilson); in some studies (Fig. 7) we spotted 20 µL per zone. Within one minute after spotting, the substrates were immersed in warm (36°C) media. The substrates with cells were incubated in the media for 24 h before stacking.

### Culture of cells in multi-layered stacks

Because there is no adhesion between layers of paper submerged in water, the layers must be held together physically (mechanical clamps) or chemically (adhesives). We could not find reliable chemical adhesives that work under water and seal the paper layers reversibly without any effect on cells. The layers were clamped using steel plates with a 12×8 layout of holes which are held together by five screws. To assist alignment of layers, each paper layer contained guiding holes for the screws. The final assembled construct fits in a standard 14 cm Petri Dish (Fig. 1C; detailed footprints of the holders are available upon request).

### Analysis of multi-zone arrays

After culture of the cells in multi-layer substrates, we opened the mechanical clamp and peeled the paper layers apart using tweezers. Typhoon FLA 9000 gel scanner (100 µm resolution, GE Healthcare) imaged the GFP and/or mTomato fluorescence signal from the cells in the paper. Intensity of the GFP or Tomato signal was converted to number of cells using calibration curves (e.g. Fig. 1F). To visualize live and dead cells, the layers were incubated in Hank's Balanced Salt Solution (HBSS) containing 4 µg/mL calcein AM and 1 µg/mL propidium iodide for 20 min. After incubation, the paper layers were rinsed three times with cold HBSS and imaged using the Typhoon. Fluorescent intensity from calcein or PI-stained samples, in principle, can also be calibrated to calculate the absolute number of live and dead cells (Fig. S5).

### Image analysis and data processing

We developed simple image processing software and graphic user interface (GUI) using MatLab™ (Fig S6). We provide a brief description of the software because it was central for analysis of all samples, and we could not find analogous integrated software packages for this purpose. The software works with images that contain multi-zone layers in arbitrary locations or orientations. (1) It takes the TIFF image recorded by fluorescent scanner or other technique (e.g. stitched-mosaic microscopy) as input. (2) The user defines the number of layers within the image and the number of the rows N and columns M in each multi-zone layer. (3) The user indicates the size of the zone (radius R) and spacing between the zones. These values can be pre-set for a standard array (e.g. 9 mm pitch and 5 mm diameter for our 96-zone layers). (4) The user clicks on the approximate locations of each multi-zone array in the image to define the top-left and bottom-right corner of the region of interest (ROI) that contains one layer. The software then divides the ROI into NxM cell grid, runs edge detection algorithm in each cell, and fits a circle with radius R to the detected edges to identify the border of the zone. Edge detection works due to a difference in auto-fluorescence of wax and paper. For most images, >98% of the circles are fit automatically to the edge of the zone. (5) Zones that were not detected accurately can be corrected by the user interactively. (6) Within detected circles, the software analyzes radial distributions of intensity (using a modified shared script of radial scan from MatLab Central: http://www.mathworks.com/matlabcentral/fileexchange/18102). It also records the intensities in the areas at 1.2*R that contains wax patterned paper and no cells. These values were used to calculate the average background intensity for the multi-zone layer. The difference between the average background intensity and average (or average radial) zone intensity is used for analysis and calibration (Fig. 1). The software saves the results as a MatLab file, which contains the coordinates and radial distributions of every zone in every layer in the input image. These intensities can be processed using separate MatLab modules to generate heat map plots, statistics, calibration, migration analysis, etc. They can be exported to other formats (tabulated text, Excel™, etc). File S1 contains MatLab code (38 scripts), test files, calibration files and detailed description of the software (Appendix 1: step-by-step instructions; Appendix 2 and 3: description of the scripts). Note: The original version of the software, included in the supporting information, has not been debugged thoroughly. Updated versions will be available upon request.

## Supporting Information

Figure S1 **Analysis of cell distribution across different types of paper.** (A) Scheme of the analysis. We prepared a suspension (10^7^ cells/mL) of MDA-MB-231-GFP cells in Matrigel (diluted with media 50∶50), and we spotted 4 µL of this suspension on 14 different types of paper (10–30 replicates). For thicker paper (marked by *), 4 µL was not sufficient to permeate through the thickness of the paper; for these papers we spotted 20 µL of suspension. We scanned the two sides of the paper using fluorescent scanner and quantified the fluorescence of cell-containing areas using image J. Panel (B) summarizes the property of each type of paper. Panel (C) contains images of the GFP fluorescence of the two sides of the same paper substrate. Plot in (D) is the difference in fluorescence intensity between the two sides (results from 8–20 measurements). Papers for which difference approaches zero have the most uniform distribution of cells throughout the paper. We concluded that papers must be thin (below 200 µm) and highly porous to allow the cells to be distributed uniformly throughout the paper. We selected paper 114 for our assays (over other, similar types) due to its exceptional stability in water.
(TIF)

Figure S2 **Raw fluorescent images of the 48-zone plates that contain MDA-MB-231-GFP cells.** (A) depicts GFP intensity in layers L1 through L8 prior to the stacking and culture as a multi-layer (see Fig. 4 and 5 for quantitative analysis). (B) describes GFP intensity in the same layers after nine days of culture. (C–F) describe the intensity of the same samples stained by various stains. (C) Incubation of the layers with calcein stains live cells. (D) Application of PI solution to unfixed cells stains only those with compromised membrane; (E) application of PI to fixed and permeabilized cells stains nuclei of all cells. (F) shows the distribution of cells that contain F-actin as detected by staining the samples by phalloidin-Alexa Fluor 633 conjugate. The distribution of cells with F-actin does not correlate with distribution of live cells as detected by GFP (B). It correlates, however, with distribution of stain in (D) (all cells).
(TIF)

Figure S3 **Migration of MDA-MD-231 cells labeled with GFP and mTomato markers.** (A–I) Heat maps describing experiments that contain 3D cultures composed of GFP and mTomato cells. (A–C) Distribution of cells in Row 1 is identical to that in Fig 4. (D–F) Distribution of cells in Row 2 is the opposite to that in row 1 (positions of GFP and mTomato cells were flipped). (G–I) In Row 3, each zone contained a 50∶50 mixture of GFP and mTomato cells. Yellow color indicates equal number of cells in zones; presence of red (e.g. layer L1) or green color (e.g. middle of layers L2–L8) indicates preferential growth of GFP or mTomato cells in these locations.
(TIF)

Figure S4 **We assembled the stacks identical to those described in** **Fig. 4** **using GFP and mTomato-labeled MDA-MB-231 cells treated with Mitomycin C (MMC).** (A) describes stacking and de-stacking of geometry e1. (B) Quantification of the fraction of the cells that migrated to the adjacent layers was performed as described in Fig. 4C (e.g. for migration from L2 to L3, it was calculated as number of cells in L2/(L2+L3)). (C) Migration depends on the relative number of cells in “sender” and “receiver” layers. D) For layers that contained similar number of cells, migration of cells was directional: significantly more cells migrated to upper layer (towards oxygen) than to the lower layer. (E) Migration of cells to the upper layer, depended on the location of the cells inside the stacks: cells in hypoxic layer L8 migrated significantly less that those in layers L1 and L5 with higher oxygen concentration.
(TIF)

Figure S5 **Analysis of arrays that contain eight different concentrations of cells (0.4×10^5^ cells to 20×10^5^ cells per zone).** We prepared a solution for MDA-MB-231-GFP cells in Matrigel and spotted 4 µL of those solutions onto the 96-zone plate. The samples were equilibrated in growth medium for three hours and then scanned with a gel scanner and a micro plate reader. (A) is the image acquired with the gel scanner; (B) is the analysis of the grey-scale intensity in cell-containing zones in image (A); (C) are results from the plate reader and (D) is the correlation of the analyses in (B) and (C). (E–L) describe characterization of the same samples after incubation with solution of calcein (E–H) or propidium iodide (PI) in the presence of Triton X-100 (I–L).
(TIF)

Figure S6 **Screen shot displaying functional modules of the custom MatLab software used for image analysis.** (A) GUI containing all the commands for image processing. (B) Interactive window that describes outlines of the zone fit by software. It allows the user to adjust positions of the zones, if necessary. (C) displays raw TIFF image that contains eight 48-zone layers (the data set is identical to that displayed in Fig. S2 (calcein), or Fig. 6E). In three analyzed layers, the zones are marked by blue dotted outlines. (D) Heat map of the average radial intensities of grey-scale distributions within the zones; the results are grouped according to experiments and replicates defined in (E). (E) depicts the window that defines how many different experiments and replicas are present in each layer. Current data set contains six experiments (1 through 6) arranged in six columns. The zones within the same column are replicates. More details are available in the code of the software (39 MatLab scripts), and brief description of the software (Supporting_Appendix_1_2.doc, Supporting_Appendix_3.doc) which are included in File S1. Detailed description of each module, troubleshooting of the software, or updates are available upon request.
(TIF)

File S1 **MatLab code (38 scripts), test files, calibration files and detailed description of the software (Appendix 1: step-by-step instructions; Appendix 2 and 3: description of the scripts).**
(RAR)
