# Supplementary figures and images for: Multizone Paper Platform for 3D Cell Cultures
Source: PLoS One. 2011 May 6;6(5):e18940. doi: 10.1371/journal.pone.0018940 (PMC3089608; doi:10.1371/journal.pone.0018940)

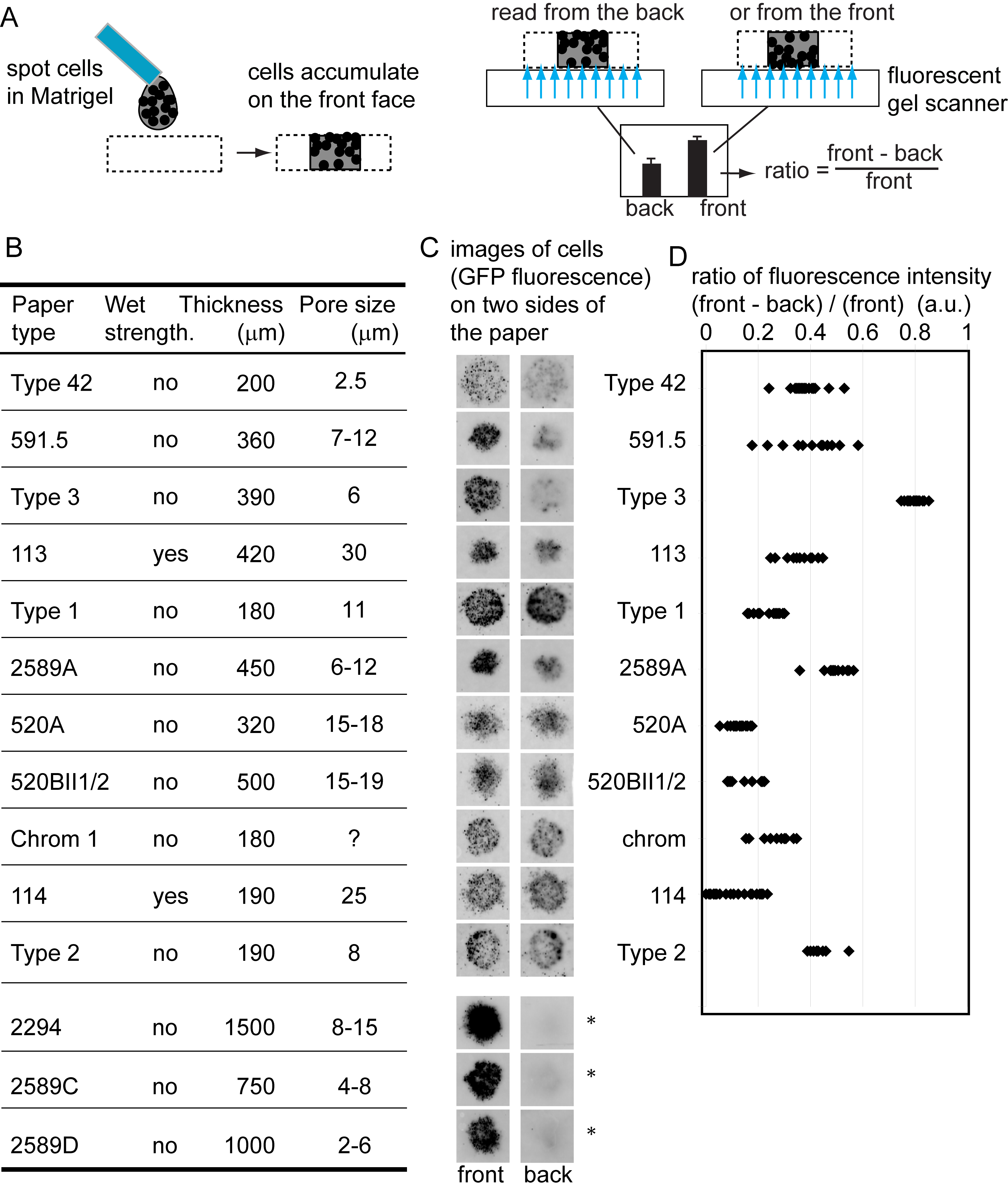

Supplement: Figure S1 — Analysis of cell distribution across different types of paper. (A) Scheme of the analysis. We prepared a suspension (107 cells/mL) of MDA-MB-231-GFP cells in Matrigel (diluted with media 50∶50), and we spotted 4 µL of this suspension on 14 different types of paper (10–30 replicates). For thicker paper (marked by *), 4 µL was not sufficient to permeate through the thickness of the paper; for these papers we spotted 20 µL of suspension. We scanned the two sides of the paper using fluorescent scanner and quantified the fluorescence of cell-containing areas using image J. Panel (B) summarizes the property of each type of paper. Panel (C) contains images of the GFP fluorescence of the two sides of the same paper substrate. Plot in (D) is the difference in fluorescence intensity between the two sides (results from 8–20 measurements). Papers for which difference approaches zero have the most uniform distribution of cells throughout the paper. We concluded that papers must be thin (below 200 µm) and highly porous to allow the cells to be distributed uniformly throughout the paper. We selected paper 114 for our assays (over other, similar types) due to its exceptional stability in water. (TIF) [file pone.0018940.s001.tif]

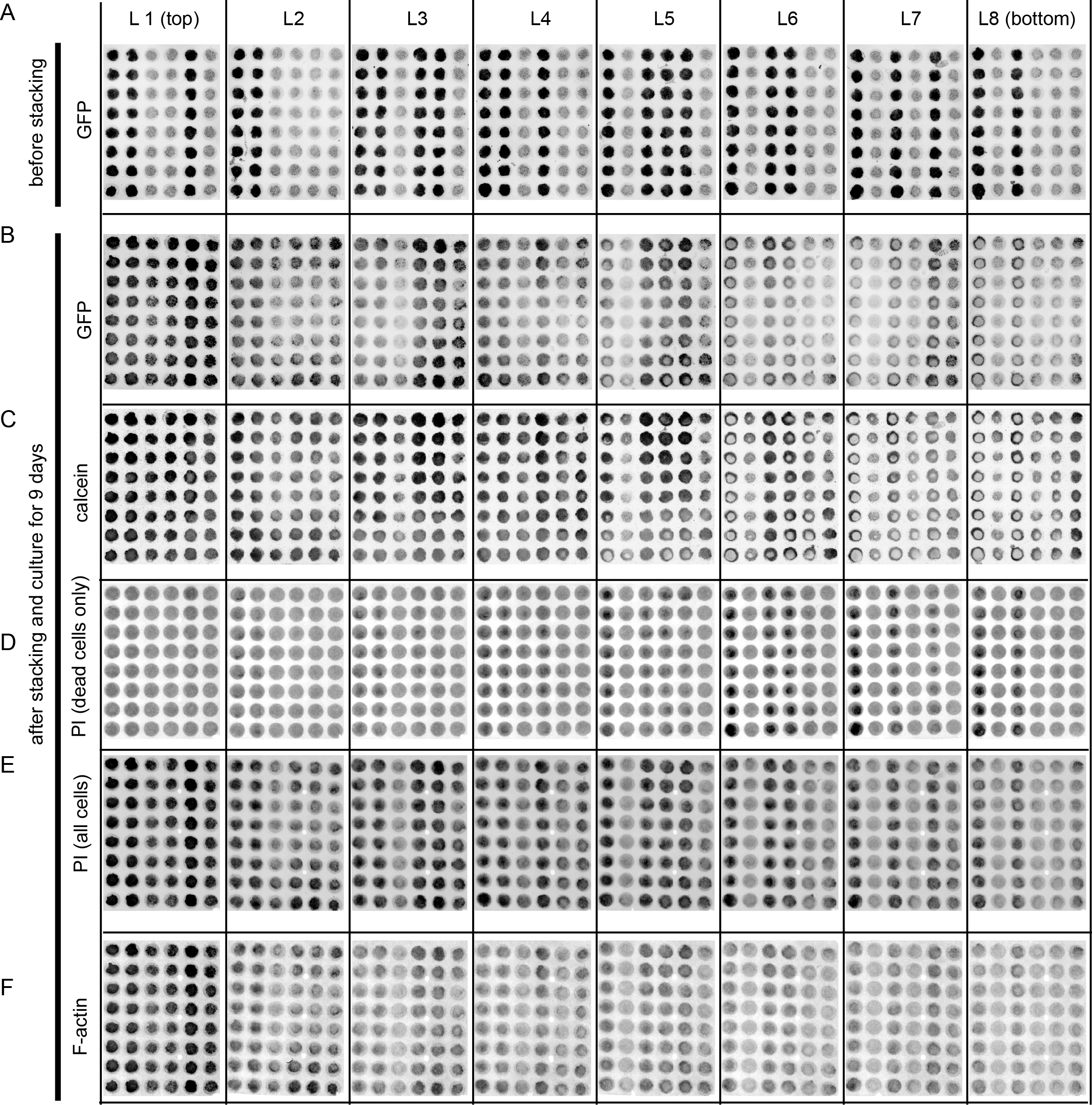

Supplement: Figure S2 — Raw fluorescent images of the 48-zone plates that contain MDA-MB-231-GFP cells. (A) depicts GFP intensity in layers L1 through L8 prior to the stacking and culture as a multi-layer (see Fig. 4 and 5 for quantitative analysis). (B) describes GFP intensity in the same layers after nine days of culture. (C–F) describe the intensity of the same samples stained by various stains. (C) Incubation of the layers with calcein stains live cells. (D) Application of PI solution to unfixed cells stains only those with compromised membrane; (E) application of PI to fixed and permeabilized cells stains nuclei of all cells. (F) shows the distribution of cells that contain F-actin as detected by staining the samples by phalloidin-Alexa Fluor 633 conjugate. The distribution of cells with F-actin does not correlate with distribution of live cells as detected by GFP (B). It correlates, however, with distribution of stain in (D) (all cells). (TIF) [file pone.0018940.s002.tif]

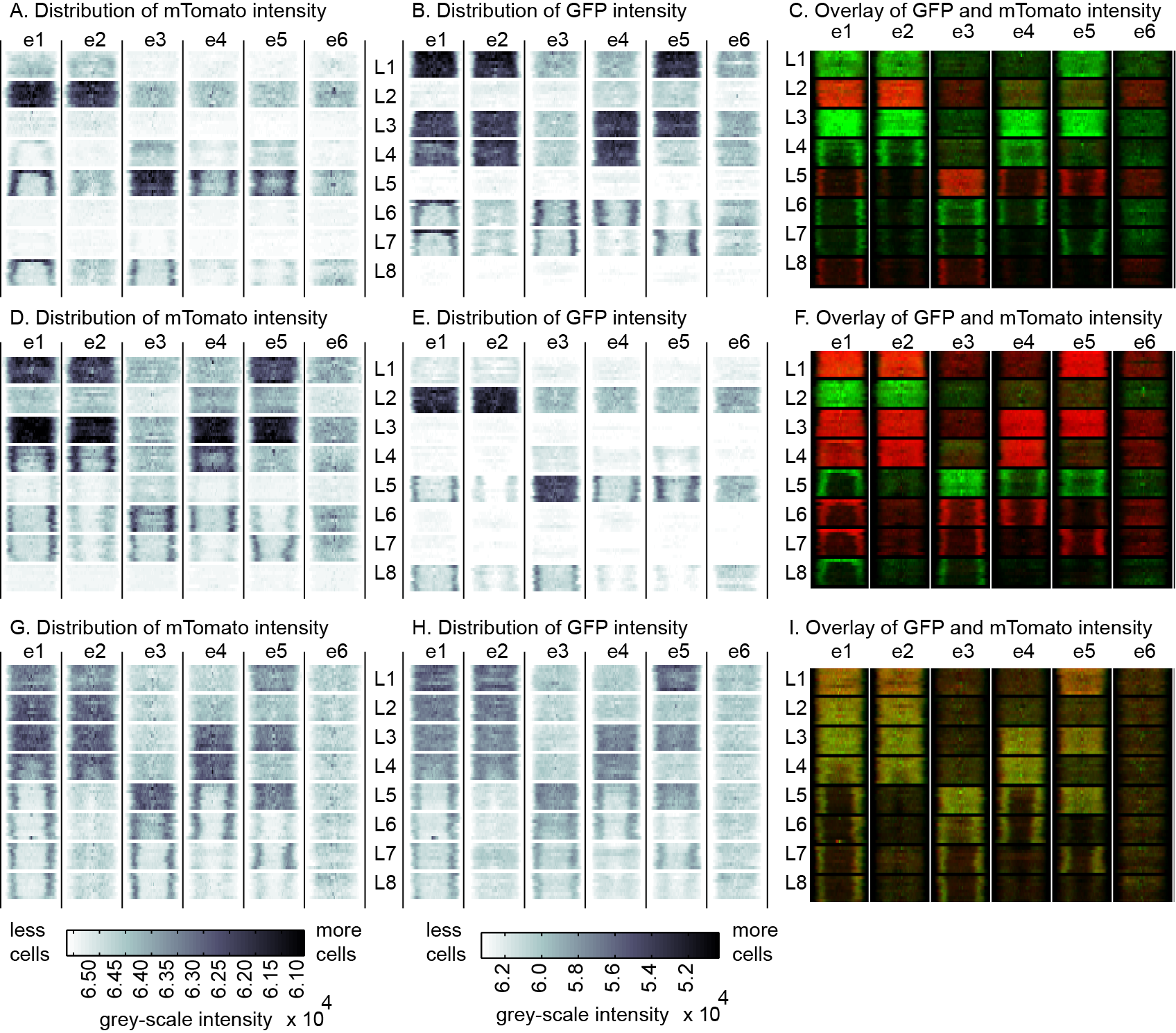

Supplement: Figure S3 — Migration of MDA-MD-231 cells labeled with GFP and mTomato markers. (A–I) Heat maps describing experiments that contain 3D cultures composed of GFP and mTomato cells. (A–C) Distribution of cells in Row 1 is identical to that in Fig 4. (D–F) Distribution of cells in Row 2 is the opposite to that in row 1 (positions of GFP and mTomato cells were flipped). (G–I) In Row 3, each zone contained a 50∶50 mixture of GFP and mTomato cells. Yellow color indicates equal number of cells in zones; presence of red (e.g. layer L1) or green color (e.g. middle of layers L2–L8) indicates preferential growth of GFP or mTomato cells in these locations. (TIF) [file pone.0018940.s003.tif]

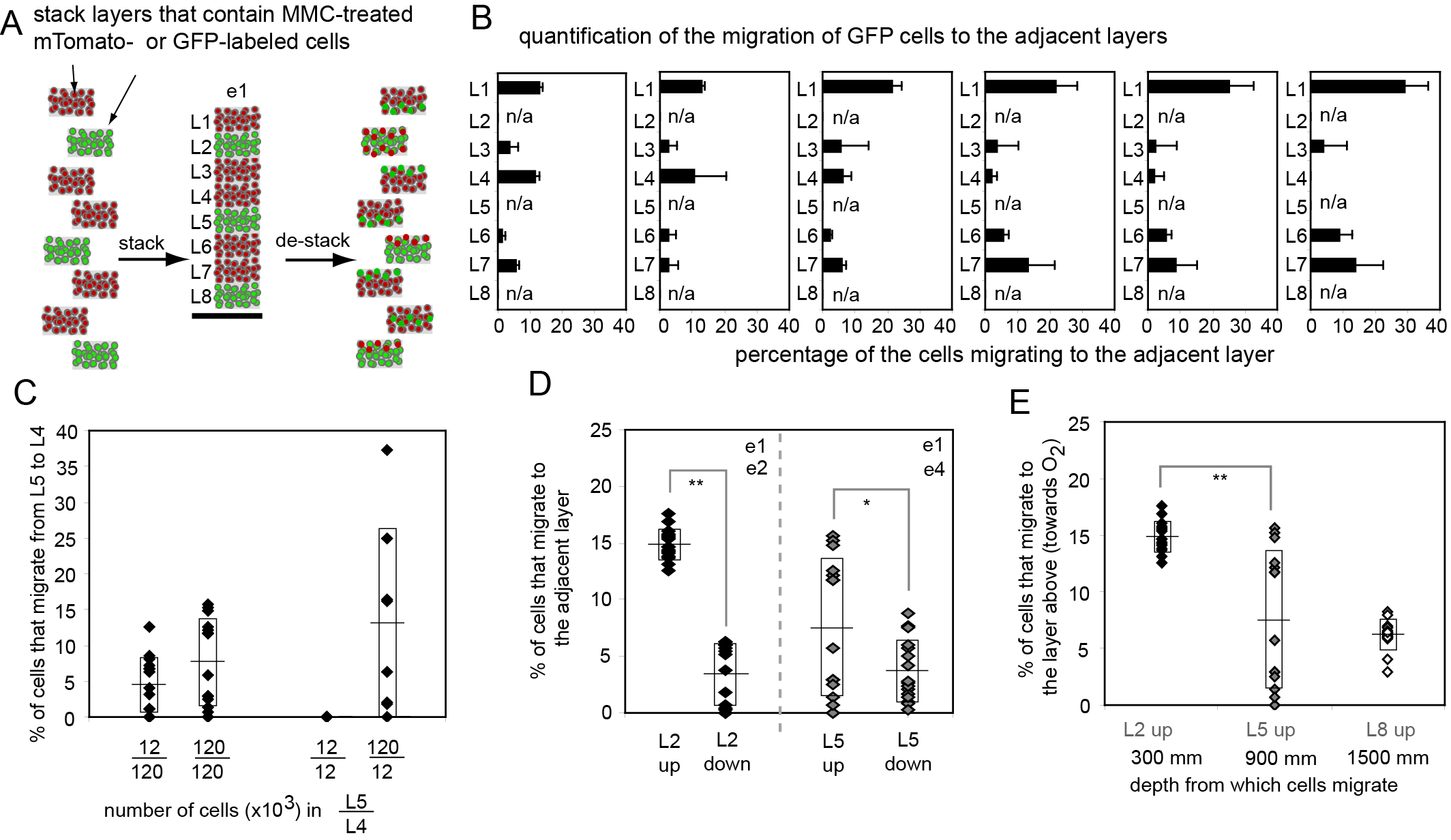

Supplement: Figure S4 — We assembled the stacks identical to those described in Fig. 4 using GFP and mTomato-labeled MDA-MB-231 cells treated with Mitomycin C (MMC). (A) describes stacking and de-stacking of geometry e1. (B) Quantification of the fraction of the cells that migrated to the adjacent layers was performed as described in Fig. 4C (e.g. for migration from L2 to L3, it was calculated as number of cells in L2/(L2+L3)). (C) Migration depends on the relative number of cells in “sender” and “receiver” layers. D) For layers that contained similar number of cells, migration of cells was directional: significantly more cells migrated to upper layer (towards oxygen) than to the lower layer. (E) Migration of cells to the upper layer, depended on the location of the cells inside the stacks: cells in hypoxic layer L8 migrated significantly less that those in layers L1 and L5 with higher oxygen concentration. (TIF) [file pone.0018940.s004.tif]

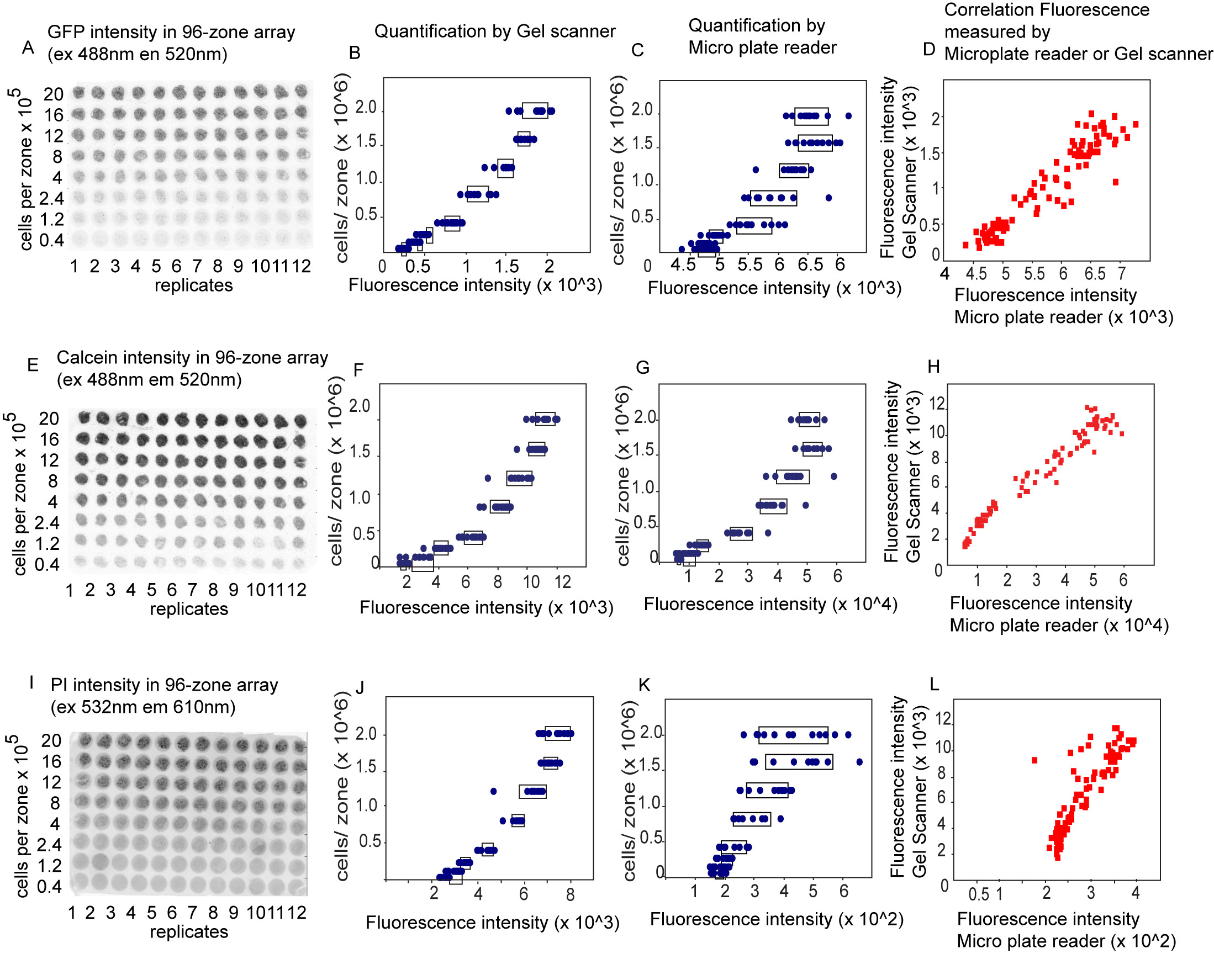

Supplement: Figure S5 — Analysis of arrays that contain eight different concentrations of cells (0.4×10^5^ cells to 20×105 cells per zone). We prepared a solution for MDA-MB-231-GFP cells in Matrigel and spotted 4 µL of those solutions onto the 96-zone plate. The samples were equilibrated in growth medium for three hours and then scanned with a gel scanner and a micro plate reader. (A) is the image acquired with the gel scanner; (B) is the analysis of the grey-scale intensity in cell-containing zones in image (A); (C) are results from the plate reader and (D) is the correlation of the analyses in (B) and (C). (E–L) describe characterization of the same samples after incubation with solution of calcein (E–H) or propidium iodide (PI) in the presence of Triton X-100 (I–L). (TIF) [file pone.0018940.s005.tif]

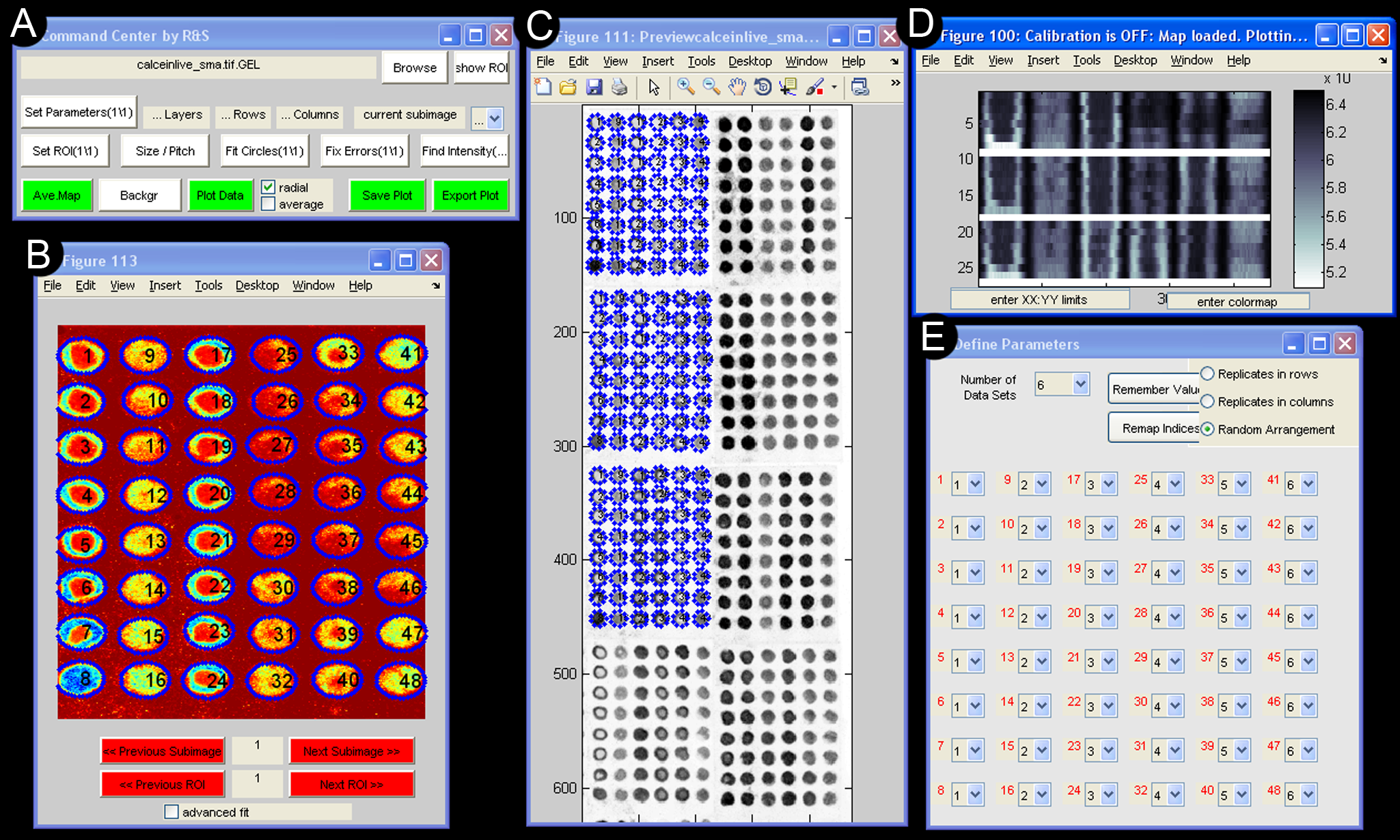

Supplement: Figure S6 — Screen shot displaying functional modules of the custom MatLab software used for image analysis. (A) GUI containing all the commands for image processing. (B) Interactive window that describes outlines of the zone fit by software. It allows the user to adjust positions of the zones, if necessary. (C) displays raw TIFF image that contains eight 48-zone layers (the data set is identical to that displayed in Fig. S2 (calcein), or Fig. 6E). In three analyzed layers, the zones are marked by blue dotted outlines. (D) Heat map of the average radial intensities of grey-scale distributions within the zones; the results are grouped according to experiments and replicates defined in (E). (E) depicts the window that defines how many different experiments and replicas are present in each layer. Current data set contains six experiments (1 through 6) arranged in six columns. The zones within the same column are replicates. More details are available in the code of the software (39 MatLab scripts), and brief description of the software (Supporting_Appendix_1_2.doc, Supporting_Appendix_3.doc) which are included in File S1. Detailed description of each module, troubleshooting of the software, or updates are available upon request. (TIF) [file pone.0018940.s006.tif]
